# Spiroacetals in the Colonization Behaviour of the Coffee Berry Borer: A ‘Push-Pull’ System

**DOI:** 10.1371/journal.pone.0111316

**Published:** 2014-11-07

**Authors:** Teresiah Nyambura Njihia, Juliana Jaramillo, Lucy Murungi, Dickson Mwenda, Benedict Orindi, Hans-Michael Poehling, Baldwyn Torto

**Affiliations:** 1 International Centre of Insect Physiology and Ecology (*icipe*), Nairobi, Kenya; 2 Department of Horticulture, Jomo Kenyatta University of Agriculture and Technology, Nairobi, Kenya; 3 Institute for Horticultural Production Systems - Plant Protection, Leibniz University Hannover, Hannover, Germany; Swedish University of Agricultural Sciences, Sweden

## Abstract

Coffee berries are known to release several volatile organic compounds, among which is the spiroacetal, conophthorin, an attractant for the coffee berry borer *Hypothenemus hampei*. Elucidating the effects of other spiroacetals released by coffee berries is critical to understanding their chemo-ecological roles in the host discrimination and colonization process of the coffee berry borer, and also for their potential use in the management of this pest. Here, we show that the coffee berry spiroacetals frontalin and 1,6-dioxaspiro [4.5] decane (referred thereafter as brocain), are also used as semiochemicals by the coffee berry borer for host colonization. Bioassays and chemical analyses showed that crowding coffee berry borers from 2 to 6 females per berry, reduced borer fecundity, which appeared to correlate with a decrease in the emission rates of conophthorin and frontalin over time. In contrast, the level of brocain did not vary significantly between borer- uninfested and infested berries. Brocain was attractive at lower doses, but repellent at higher doses while frontalin alone or in a blend was critical for avoidance. Field assays with a commercial attractant comprising a mixture of ethanol and methanol (1∶1), combined with frontalin, confirmed the repellent effect of this compound by disrupting capture rates of *H. hampei* females by 77% in a coffee plantation. Overall, our results suggest that the levels of frontalin and conophthorin released by coffee berries determine the host colonization behaviour of *H. hampei*, possibly through a ‘push-pull’ system, whereby frontalin acts as the ‘push’ (repellent) and conophthorin acting as the ‘pull’ (attractant). Furthermore, our results reveal the potential use of frontalin as a repellent for management of this coffee pest.

## Introduction

The coffee berry borer (*Hypothenemus hampei*) [Scolytinae: Coleoptera] is the most important and damaging pest of commercial coffee [1], causing losses greater than US$ 500 million annually, and threatening the livelihoods of 100 million small-holder farmers worldwide. Given the cryptic nature of the pest [2], non-selective pesticides provide the main method of effective control [3]. However, the development of insecticide resistance in the pest and the growing environmental concerns of synthetic chemical pollutants [4], [5] has led to the search for alternative sustainable control strategies for the pest including the use of semiochemicals [1]. An example of a semiochemical that has been tested as a mass-trapping lure for the coffee berry borer with limited success is a 1∶1 mixture of methanol and ethanol. To improve upon this, a better understanding and exploitation of the behaviour and chemical ecology of the coffee berry borer is required. For example, in previous behavioural studies, while females were attracted to healthy ripe coffee berries, borer-infested berries repelled them [6]. Also, colonizing females were found to hardly share a host [1], [7]. These observations suggest the involvement of a female host marking pheromone (HMP) or berry-induced defensive/repellent compounds affecting conspecifics. In scolytinae species, this characteristic behaviour is commonly referred to as ‘maximum attack density’ (MAD), and it involves attack on a unit area of a host in a uniform pattern to enhance spacing for reproduction by individual pest during the host colonization process [8]–[10]. In some scolytid species, once the host's threshold population has been reached, then allomones and pheromones are produced to signal to conspecifics to search for healthy hosts. Therefore, it is desirable to explore whether a similar interaction occurs between the coffee berry borer and its coffee berry host.

Previous studies suggested that host finding in the coffee berry borer is regulated by olfactory cues and several coffee berry volatile organic compounds were identified as semiochemicals for the borer [11]–[12]. Among these are chalcogran, verbenone, α-pinene, and the spiroacetals conophthorin, 1,6 dioxaspiro [4.5] decane (brocain) and frontalin. Of the three spiroacetals, frontalin is a multifunctional pheromone particularly amongst *Dendroctonus spp*; as a spacer, and both an aggregation and an anti-aggregation pheromone as well as a sex pheromone [13]–[17]. While conophthorin is an attractant for the coffee berry borer [12], the behavioural significance of 1,6-dioxaspiro [4.5] decane (brocain) and frontalin, both of which elicit antennal activity in the insect [12] is unknown. We have expanded on these findings and here we present results of laboratory and field studies on the effect of these two specific spiroacetals in the host colonization process by *H. hampei*, with emphasis on their potential use in the management of this pest.

## Materials and Methods

### Insects

Females of the coffee berry borer used in the experiments were obtained from a colony maintained in the laboratories of the International Centre of Insect Physiology and Ecology (*icipe*), Duduville campus, Nairobi, Kenya. The insects were reared using a technique developed by Jaramillo et al. [18], which utilizes fresh coffee berries in order to closely mimic field conditions. The borers were reared on 150 days old coffee berries (*C. arabica* var. Ruiru 11) collected from a plot in a privately owned coffee plantation in Kiambu (Central province), Kenya. The colony was kept at room temperature (25±1°C), 70%±5% relative humidity [RH], and a 12∶12 h (L: D) photoperiod. Infested berries were kept inside square plastic containers (40 x 40 x 20 cm) with perforated lids (55 mm diameter) covered with insect gauze. The bottom of each container was layered with a 2 cm mixture of plaster of Paris and activated charcoal to maintain humidity and to prevent the desiccation of berries and insects.

### Study site

Laboratory experiments were carried out at the International Centre of Insect Physiology and Ecology (*icipe*), Duduville campus, Nairobi, while field tests were carried out in Kiambu, Central Province of Kenya (1° 11′ 27.15″ S; 36° 49′ 23.03″E. altitude 1,722 m.a.s.l). No specific permissions were required for field study/collections. The field studies did not involve endangered or protected species. The owner of the land gave permission to conduct the study on this site.

### Coffee berries

For all the experiments, organically grown coffee berries (*Coffea arabica* L. var. Ruiru 11) were collected from Kiambu district, Central Kenya (see above). Berries of approximately 150 days old (yellow/orange exocarp stage), the most attractive developmental stage to *H. hampei* [19], were randomly sampled in the field. They were excised directly from coffee tree branches without hand contact, using a sterile scalpel blade No. 21.

### Chemicals

Authentic standards of frontalin (1,5-dimethyl–6,8-dioxabicyclo[3.2.1]octane), purity ≥98%, was purchased from ConTech Inc. (USA); (*5S,7S*)-conophthorin, and 1,6- Dioxaspiro[4.5]decane (herein called brocain) (purity 97%) was provided by Prof. Wittko Francke, University of Hamburg, Germany.

### Preliminary laboratory experiments

For *H. hampei* berry infestation, four different stages of attack are recognized [20]. (A), onset of female colonization of a new berry but without penetration of the exocarp; (B), penetration of the berry exocarp, but without reaching the endosperm; (C), boring into the endosperm and gallery construction with no oviposition; and (D), gallery construction accomplished, oviposition, and one or more immature stages present inside the gallery. In this study, we observed that *H. hampei* females spent approx. 1–2 hrs, 6–8 hrs, 10–12 hrs and 6–15 days to attain infestation stages A, B, C and D respectively.

To investigate the volatile composition associated with coffee berries in the different stages of infestation by *H. hampei* females, we carried out preliminary assays as follows: Field-collected, infested and uninfested coffee berries in the orange-red exocarp stage (see above), were placed in separate sterile cylindrical glass jars (0.5 L) with single-port lids (Analytical Research Systems INC, Gainesville, FL, USA) and transported to the laboratory. Headspace volatiles were collected from both the uninfested (101 berries, ∼170 g) and berries (50 berries, ∼83 g) in advanced infestation stage. Two equal aliquots were prepared from the -uninfested coffee berry sample, of which one was subjected to headspace volatile collection and the other artificially infested with three *H. hampei* females per berry. Infestation stages B and C were obtained after 10 hrs of infestation, as described above. Volatiles were collected from this sample as well as frass (∼3 g).

GC-MS analyses of the volatiles of the above samples identified (*5S,7S*)-conophthorin as the major component (figure S1), with frontalin detected in relatively lower levels (figure S1). In subsequent analysis, (5S, 7S)-conophthorin and frontalin were identified at varying levels as *H. hampei* infestation advanced inside the berries, with the least levels detected in the frass. These results guided experimentation to investigate the two spiroacetals emission rates as a function of the number of initial colonizing females infesting each coffee berry, different infestation stages and the number of life stages inside berries as described below.

### *H. hampei* life stages versus spiroacetals produced by berries infested with different colonizing numbers of *H. hampei* females

To investigate the volatile composition associated with coffee berries in different stages of infestation by different numbers of colonizing *H. hampei* females, randomly sampled and field-collected uninfested berries in the yellow-orange exocarp stage were checked for infestation. Only uninfested berries of uniform shape and weight were used for subsequent experiments. A total of 1200 berries were used. The berries were divided into 3 groups, (400/group) and placed in round plastic containers (23 cm diameter ×6.8 cm depth) and exposed to different numbers of *H. hampei* females: **group 1:** two *H. hampei* females per berry (total number of females, 800); **group 2:** four *H. hampei* females per berry (total number of females, 1600); **group 3:** six *H. hampei* females berry (total number of females, 2400). After infestation, the different berry groups were transferred into different rearing containers, and kept in temperature-controlled climate chambers (SANYO MIR-553, Sanyo Electrical Ltd., Japan) set at 26°C, 65% RH, and a 12∶12 h (L: D) photoperiod. To maintain the humidity inside the containers, distilled sterile water was added every three days.

Half of the berries in each group (200 berries) were kept for dissections and counting of the different *H. hampei* life stages inside them. Volatiles were collected from the other half of the berries (200). Both, the dissections and headspace volatile collections for each group were carried out after 2, 5, 15 and 30 days after the initial infestation of the berries.

For the dissections, 48 berries per evaluation time were selected (8 berries per 6 replicates) were destructively sampled under the stereomicroscope. The numbers of live and dead *H. hampei* colonizing females as well as their infestation stage (A, B, C and D) and number of beetle life stages (i.e., eggs, larvae, pupae and adults) were assessed.

### Headspace volatile collection

Headspace volatiles were collected from the *H. hampei* infested coffee berries for each of the *H. hampei* stages of infestation inside the berry, 2, 5, 15 and 30 days after the initial infestation for each group (2, 4 and 6 *H. hampei* females/berry). Volatiles were collected for 36 hr from each odour source by aeration and adsorption on pre-cleaned (methanol, dichloromethane, pentane, nitrogen-dried) charcoal filters (5 mg; Part No. 91006015; Brechbühler, Schlierensee, Switzerland). Each filter was connected by PVC tubing (Masteflex. 06409-15 Tygon mfg by St. Gobain) to a mobile battery operated pump (PAS-500 Personal Air Sampler, Supelco, Bellefonte, PA, USA), which pulled volatiles through the filter at a flow rate of 348 ml/min. All the filters were eluted with 100 µL of GC-grade dichloromethane (Sigma Aldrich, Gillingham, UK), and the eluents were stored at −80°C in a 200 µl microtube vial insert placed inside a 1.5 ml glass vial (Sun Sri, TN, USA) with a PTFE lined cap prior to analysis.

### Analysis of volatiles

Volatile extracts were analysed using coupled gas chromatography-mass spectrometry (GC-MS) on an Agilent Technologies 7890A GC linked to a 5795C MS, equipped with MSD ChemStation E.02.00.493, and Wiley 9^th^/NIST 2008 MS library and a HP5 MS column (30 m x 0.25 mm iD). The temperature program was 5 min at 35°C, then 10°C/min to 280°C. An aliquot (1 µl) of each volatile extract was analysed in the splitless mode, using helium as a carrier gas at a flow rate of 1.0 ml/min. Spectra were recorded at 70 eV in the electron impact (EI) ionisation mode, and emission current of 34.6 µA. Compounds in the volatiles were identified by comparing their mass spectra with those in the library (NIST/EPA/NIH Mass spectral Library 2005a version V2.od). Unambiguous structure assignments were based on co-injection with authentic standards.

### Behavioural assays

Behavioural responses of *H. hampei* were tested in three different experiments, comprising a Y-tube olfactometer with an air-flow, and a Petri-dish arena in still air, both carried out in the laboratory, and in field trials as follows:

#### a) Experiment 1: Y- tube Olfactometer

This assay was carried out in a Pyrex glass Y-tube olfactometer (10 mm i.d; stem 85 mm; arms 75 mm at a 60° angle to the stem) (Analytical Research Systems INC, Gainesville, FL, USA). Solutions for each compound were formulated in dichloromethane to dissolve the chemicals. The Y-arms of the olfactometer were attached with PVC tubing (Masteflex. 06409-15 Tygon, St. Gobain) to a sealed glass odour source chamber (internal volume 50 ml) supplied with charcoal-filtered and humidified air (90% RH). The airflow through each arm of the Y-tube was maintained at 100 ml/min by the positive pressure of a battery-powered pump (USDA/ARS-CMAVE, Gainesville, Fl, USA). Bioassays were carried out in a room (25±1°C; 60±5% RH) with diffused uniform fluorescent light (58 W). The assays were run only between 10:00 and 17:00 hrs to coincide with the peak of *H. hampei* female activity in the field [12]. Females of *H. hampei* used in this experiment were collected from berries infested for between 8 and 12 weeks. Prior to assays, the females were starved for 12 hrs and subsequently individually introduced into the stem of the Y-tube olfactometer. They were considered to have made a positive response after spending at least 15 sec beyond the Y-tube intersection into the arm with the tested chemical. *H. hampei* females that failed to choose an arm in 15 min were recorded as non-responders.

The Y-tube olfactory tests were carried out with the volatile extract obtained from coffee berries infested with varying number of colonizing females and at different infestation stages (see, group 1–3 above). In total, each odour source was tested in 10 replicates, with 15 *H. hampei* females individually tested per run. In addition, authentic standards of brocain and frontalin were tested in dose-response tests. The composition of blends and doses tested are listed in table 1. The blends were formulated using 40 ng/µl brocain and 5 ng/µl frontalin (blend A) as the reference blend. An initial bioassay with the individual spiroacetals showed that these two doses were the most attractive to the coffee berry borer. Varying high amounts of both compounds in the blend were also tested. Subsequently, we retained the dose of brocain but increased the amount of frontalin with 5 replicates using 15 insects/run (N = 75).

**Table 1:** Compounds and doses tested in laboratory and field experiments.

Experiment	Compounds tested	Concentration (ng/µl)	Volume
**I. Laboratory tests**			
a) Y-tube olfactometer	1. Brocain	2.5, 5, 10, 20, 40, 80,160,320, 640	40 µl
	2. Frontalin	2.5, 5, 10, 20, 40, 80, 160,320, 640	40 µl
	3. Brocain + frontalin mixtures	a) 40+5, 160+20, 640+80	40 µl
		b) 40+5, 40+10, 40+20, 40+40	40 µl
b) Petri dish	1. Brocain	40	100 µl
	2. Frontalin	80	100 µl
**II. Field trials (Plots A, B and C)**	1. Brocain	320	0.96 ml
	2. Frontalin	320	0.96 ml
	3. 1∶1 Ethanol+ methanol mixture (EM)	-	3 ml
	4. Brocain+ EM	320	0.96 ml+3 ml
	5. Frontalin + EM	320	0.96 ml+3 ml
	6. 95% distilled water +5% dimethyl sulfoxide	-	3 ml
	7. Dimethyl sulfoxide (solvent) + EM	-	0.96 ml+3 ml

An aliqout (40 µl) of the volatile extract or solvent was applied to a filter paper strip (25 x 25 mm) (No. 1 Whatman Int Ltd. Maidstone, England) and tested in assays. After solvent evaporation for 2 min the treatment and control filter paper strips were held in glass chambers (internal volume 50 ml). At intervals of 30 min, the treatment and control filter paper strips were replaced with fresh ones in order to minimize variability of odour perception among insects introduced at various times. Additionally, assignment of odour source to each arm of the olfactometer was reversed in between tests to eliminate directional bias. Blends were also tested similarly, except that 40 µl of each component was loaded on to a separate filter paper held in in the same glass chamber. The Y-tubes were exchanged with clean ones after each test and all glassware was washed with Teepol (multipurpose detergent. Teepol products, Kent, UK), rinsed with acetone and then with distilled water and baked in an oven at 100°C for 2 hr.

#### b) Experiment 2: Petri dish bioassay

This experiment was carried out to investigate *H. hampei* females infestation levels in coffee berries with brocain or frontalin applied on the exocarp in a glass Petri dish (14 cm diameter) arena, lined with filter paper (No. 1 Whatman Int. Ltd. Maidstone, England), divided into two equal halves. Responses of coffee berry borer females, released in groups into a circular area of ∼1.5 cm diameter located in the middle of the filter paper were compared between; brocain and control (solvent only), and frontalin and control (solvent only). Five berries treated with either of the compounds or solvent were placed on each half on the extreme end, equidistant from the centre and separated 9 cm apart. The experiment was run for 4 hrs, with 10 insects to allow for infestation to occur, after which the experiment was stopped. The Petri dish was rotated 90° after every 15 min to minimize positional bias and replacing the filter paper after each test. There were 10 replicates for all the different infestation days (N = 100 insects).

Subsequently, *H. hampei* infestation levels of brocain, frontalin and control (solvent) treated berries were compared jointly by dividing the Petri dish arena into three equal sections. Three berries, separately treated with the test materials, were held in each section of the Petri dish. Nine females were released in a group into the middle and their response time in the different sections recorded after 4 hrs. The Petri dish was rotated horizontally at an angle of 120° after every 15 min to exchange positions of the treatments, with 11 replicates (N = 99). Likewise, the filter paper was renewed after each test. Infestation was considered successful when at least 90% of the insects had fully penetrated the berries. In both tests, 100 µl of brocain and frontalin or solvent (table 1) were applied on to the berries.

### Field trials

We evaluated in a short field trial between 18^th^ November 2013 and 9^th^ December 2013, in the same coffee plantation where the berries for the laboratory experiments were collected, traps baited with brocain and frontalin tested alone or combined with the commercial attractant (1∶1 mixture of methanol and ethanol). No crop sanitation was carried out during the time of the field-testing in order to have an undisturbed *H. hampei* population in the target plots.

The traps were deployed in three unshaded coffee plots within the plantation, each plot with approx. 100 trees (planting density 2 x 2 m). The specific characteristics of the plots were: plot A was adjacent to a shaded plot with 15 shade trees (12 grevillea (*Grevillea robusta*), 2 avocado (*Persea americana*) and 1 mango (*Mangifera indica*); plot B was next to plot A, and plot C was at the farthest end from the shaded plot (full sunlight exposure). The spiroacetals were prepared from a stock solution (SL) of 1 mg/ml of either brocain or frontalin formulated in 5% DMSO+95% H_2_0 (solvent). Brocap traps were used to dispense the seven treatments (table 1), placed in each plot, hanging from coffee trees at approx. 120 cm above the ground and>10 m apart, with 3 replicates of each treatment over time. The compounds were placed in dispensers provided together with the traps. A uniform hole (2 mm diameter) was drilled in the dispenser. In the collection bottle of the trap, 500 ml of distilled water plus 5 drops of teepol detergent was added, to kill captured females. Treatments were replaced with new ones weekly while trapped insects were counted and held in vials with 70% ethanol for preservation. The treatments that remained in the traps' dispensers were transported to the laboratory under cool conditions (−4°C) for weighing and calculating the release rates of the different treatments whose averages are provided in table S1. Re-randomization of traps in each plot was done during each visit. Methanol: ethanol and 5% DMSO +95% H_2_0 solvent were used as positive and negative controls respectively.

### Statistical analysis

In order to standardize the outcome, the counts of lifestages (e.g. number of eggs, larvae, pupae, and adults) observed inside coffee berries were divided by the number of initial colonizing females introduced into a berry. Thereafter, the data on densities of life stages inside coffee berries over evaluation time were analysed using Analysis of Variance (ANOVA) following a Box-Cox transformation with lambda equal −0.55. Prior to transformation, we added a constant 0.01 to make the values positive. The factors in the ANOVA model were the main effects for life stage, evaluation time, crowding and their interaction terms. R version 3.1.0 [21] was used for this section.

The number of entries into treated and control arms of the olfactometer per odour source in experiments of berries infested with different numbers of *H. hampei* were compared using Chi square test. Similarly, data on olfactometer trials using authentic standards of frontalin, brocain, blends and petri dish trials were also analysed using Chi square test [22]. The numbers of coffee berry borer making choices between treatment and control, relative to the total number of insects introduced including those that did not respond, were compared using Chi Square test.

Data on field trials were analysed using Analysis of Variance (ANOVA) in SAS version 9.2 [22]. Averages of *H. hampei* females captured were log transformed before analysis using the formula log (number of catches+1). Multiple comparisons for the plots and treatments baited in traps were performed using Student-Newman-Keuls (SNK) procedure. All tests were performed at 5% significance level.

## Results

### *H. hampei* life stages in berries infested with different numbers of colonizing females

**Figure 1:**
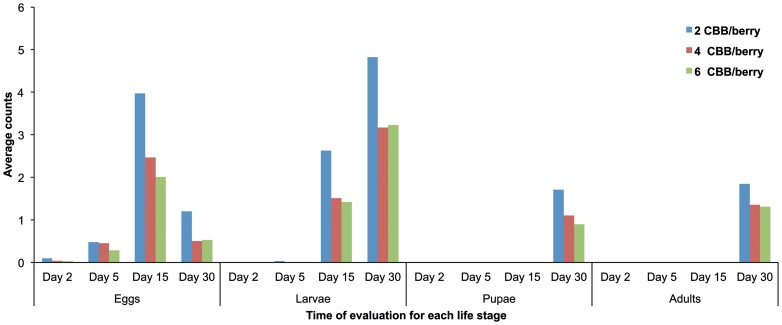
Number of *Hypothenemus hampei* (CBB) life stages found inside coffee berries (approx. 150 days of development), infested with 2, 4, and 6 *H. hampei* colonizing females per berry, after 2, 5, 15 and 30 days after infestation.

The ANOVA results indicated that the coffee berry borer life stages, evaluation time and crowding interaction were significant (*F*_18, 2256_ = 1.89, *P* = 0.0120). The effect of evaluation time and crowding within berries for each life stage was also significant for all the four life stages: eggs (*F*_6, 564_ = 3.36, *P* = 0.0029), larvae (*F*_ 6, 564_ = 2.77, *P* = 0.0115), pupae (*F*_ 6, 564_ = 6.23, *P* = <0.0001, and adult (*F_6_*_, 564_ = 2.79, *P* = 0.0110). As a consequence, we studied the effect of crowding at each evaluation time within each life stage. The results are summarized in table S2, and presented in figure 1.

### Effect of *H. Hampei* infested coffee berries on spiroacetals emission and behaviour of *H. hampei*

**Figure 2:**
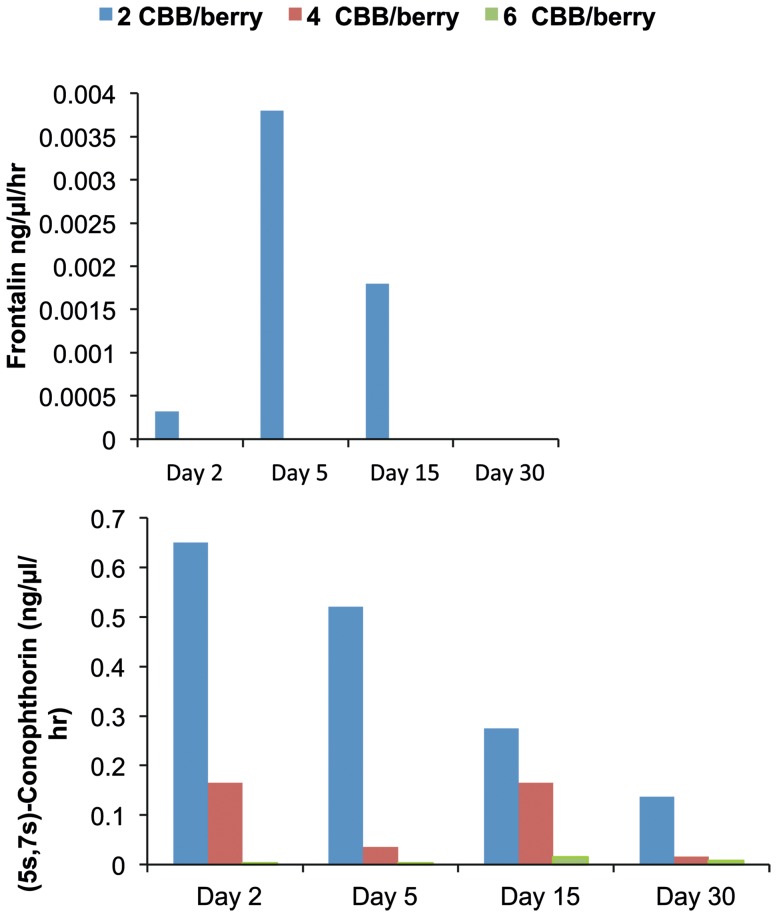
Release rate (ng/µl/hr) of (*5S,7S*)-conophthorin and frontalin in samples of coffee berries (approx. 150 days of development), infested with 2, 4 and 6 *Hypothenemus hampei* colonizing females per berry (CBB), after 2, 5, 15 and 30 days after infestation.

We assessed how *H. hampei* infestation level influenced the emission patterns of the two spiroacetals (*5S,7S*)-conophthorin and frontalin in coffee berries because of their behavioural importance [12] and how this affected the response of recipient females in subsequent assays. Notably, the berries infested by 2 initial *H. hampei* females released the highest amount of (5S, 7S)-conophthorin while those infested by 6 colonizing females released the lowest amount. Generally, conophthorin release levels diminished with increasing number of colonizing females and advancing infestation stages (figure 2). Only berries infested by two borers released frontalin – with a larger proportion released in the initial infestation stages, which decreased over time to undetectable levels after 30 days (figure 2).

**Figure 3:**
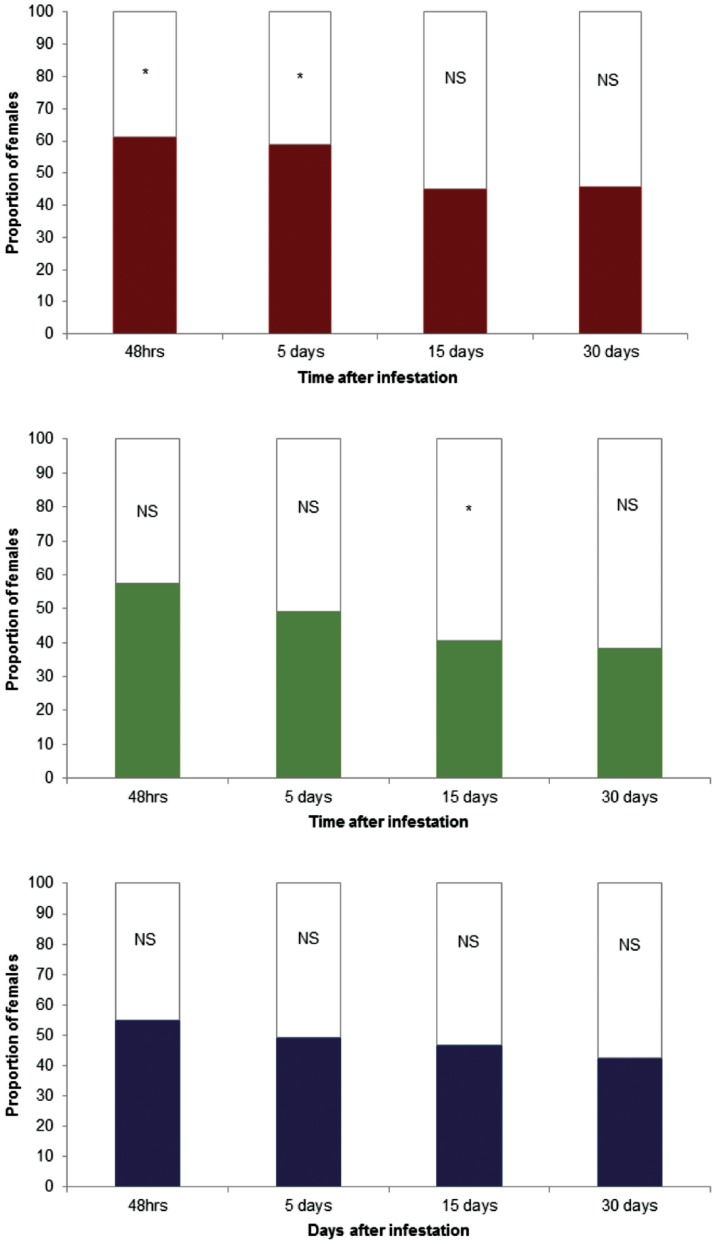
Responses of individual *Hypothenemus hampei* females (75 individuals per group/time after infestation) in olfactometer trials. Red: coffee berries infested with 2 *H. hampei* colonizing females per berry (group 1); Green: coffee berries infested with 4 *H. hampei* colonizing females per berry (group 2); Blue: coffee berries infested with six *H. hampei* colonizing females per berry (group 3).

In behavioural experiments, in the absence of odour sources, the majority of *H. hampei* females (N = 69 out of 75) failed to respond after 15 min into either arm of the olfactometer. Of the three different colonizing groups of females, headspace volatiles from the different stages was significantly attractive, only in berries infested with 2 *H. hampei* initial females/berry at 2 and 5 days post-infection (61.3%; χ^2^_1_ = 7.0146 *P* = 0.0081; and 58.7%; χ^2^_1_ = 3.8413 *P* = 0.049, respectively). However, no significant behavioural response was recorded for all the groups, at 15 and 30 days after infestation, except for group 2 (4 *H. hampei/*berry), which was significantly avoided at 15 days (59.4% for blank; χ^2^_1_ = 4.6992 *P* = 0.0302) (figure 3).

### Behavioural assays with authentic chemicals

To elucidate the role of frontalin and the spiroacetal 1,6-dioxaspiro[4.5] decane (brocain) in the colonization process of *H. hampei*, we tested these compounds in different doses alone and in mixtures using authentic standards.

**Figure 4:**
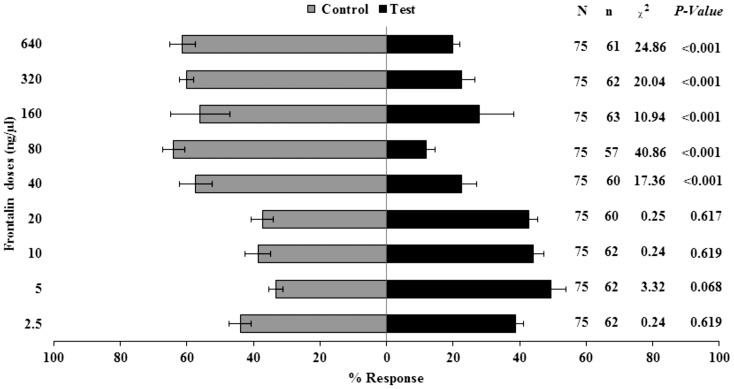
Olfactometer responses of *Hypothenemus hampei* females to frontalin (mean ± SE). N =  total number of insects, and n =  total respondents (i.e. n = N less non-respondents)). Positive response to the various concentration levels is referred to as test while responses to DCM solvent is the control. The percent response for each arm was calculated relative to N.

No significant differences were found between frontalin and control (solvent) for the first four least doses (2.5, 5, 10, 20 ng/µl) (figure 4). Among the doses tested, 5 ng/µl was the most attractive (+16% of attraction more than control), although not significant (P = >0.05). All doses above 40 ng/µl were significantly repellent to coffee berry borer females. 80 ng/µl frontalin was the optimal dose with only 12% of insects preferring it and 64% opting for the control (figure 4).

**Figure 5:**
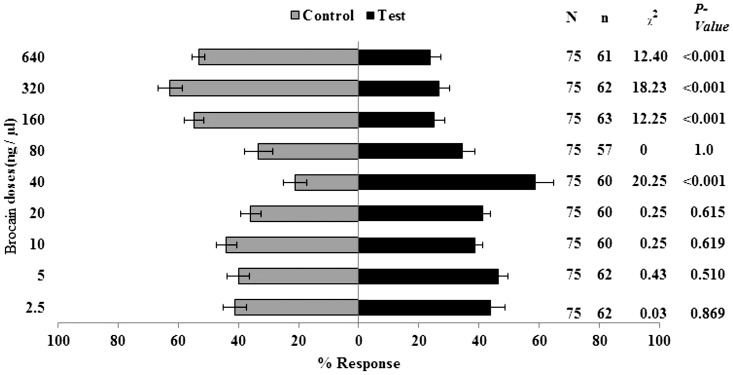
Olfactometer responses of *Hypothenemus hampei* females to brocain (mean ± SE). N =  total number of insects, and n =  total respondents. Positive response to various concentration levels is referred to as test while to DCM solvent as control. The percent response for each arm was calculated relative to N.

Likewise, as observed for frontalin, responses to doses between 2.5 and 20 ng/ul of brocain were not significantly different (figure 5). However, at 40 ng/µl of brocain there was a threefold (58%) increase in the number of coffee berry borers preferring the treatment compared to the control (21%). No significant responses were observed for 80 ng/µl. However, all doses above 160 ng/µl were avoided by borers, which preferred the solvent (figure 5).

**Figure 6:**
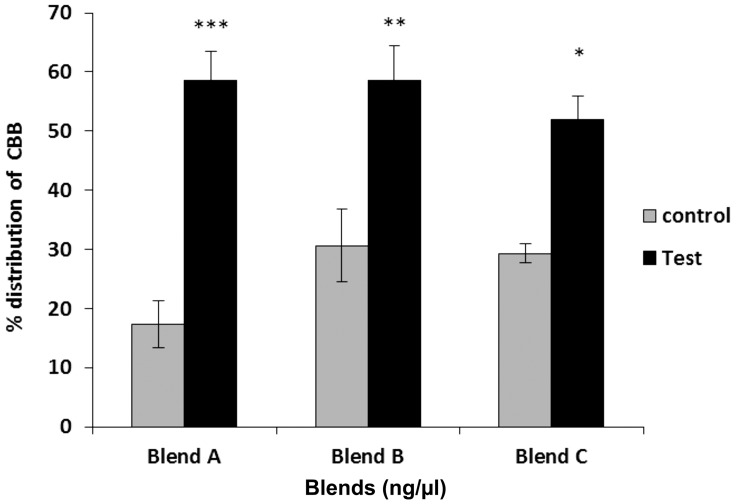
Olfactometer responses of *Hypothenemus hampei* females (CBB) to blends (mean ± SE). Blend A: 40 ng/µl brocain +5 ng/µl frontalin, blend B: 160 ng/µl brocain +20 ng/µl frontalin, blend C: 640 ng/µl brocain +80 ng/µl frontalin. The asterisks indicate the significance levels (* =  significant at 0.05, ** =  significant at 0.01 and *** =  significant at 0.001).

Blends A (40 ng/µl+5 ng/µl), B (160 ng/µl+20 ng/µl) and C (640 ng/µl+80 ng/µl) of brocain and frontalin respectively (figure 6) were significantly more attractive to *H. hampei* females (blend A: +41%, χ^2^_1_ =  25.47, P =  < 0.001; blend B: +28%, χ^2^_1_ =  10.79, P  = < 0.01; and blend C: +22.7%, χ^2^_1_ =  7.07, P  = < 0.05) than control (figure 6). Although the amounts of brocain and frontalin in the blends were increased, their ratios in the blends were constant (8∶1). Notably, blend B and blend C partially or fully comprised doses that were individually avoided by the borers (figures 4 and 5), when presented in a blend were attractive. However, increasing blend dose significantly reduced borer attraction. The ranking of preference by *H. hampei* females follows the order blend A>blend B> blend C (figure 6).

**Figure 7:**
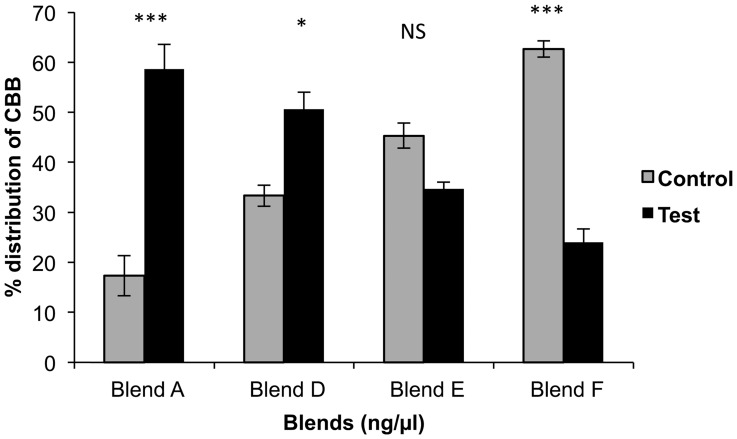
Olfactometer responses of *Hypothenemus hampei* females (CBB) to blends (mean ± SE). Blend A: 40 ng/µl brocain +5 ng/µl frontalin; blend D: 40 ng/µl brocain +10 ng/µl frontalin; blend E: 40 ng/µl brocain +20 ng/µl frontalin; and blend F: 40 ng/µl brocain +40 ng/µl frontalin. The asterisks indicate the significance levels (* =  significant at 0.05 and *** =  significant at 0.001).

In follow up tests, 40 ng/µl brocain was used in the formulations of all the blends but the amounts of frontalin in the blends varied to comprise 5, 10, 20 and 40 ng/µl, respectively. Addition of frontalin to the blends constantly reduced *H. hampei* attractiveness to the blends (blend A: +41%, χ^2^_1_ =  25.47, P =  < 0.001; blend D: +17%, χ^2^_1_ =  3.94, P =  < 0.05; and blend E: +10.7%, χ^2^_1_ =  1.36, P =  > 0.05) (figure 7). Blend F that comprised the highest amount of frontalin significantly repelled the borers (blend X: −38%, χ^2^_1_ =  21.29, P  = < 0.001) (figure 7).

**Figure 8:**
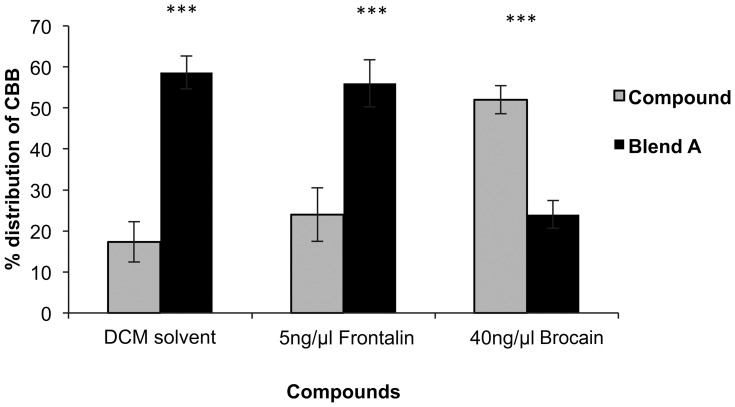
Comparison of *Hypothenemus hampei* (CBB) responses to an optimal blend (A) against DCM solvents and individual components of the blend, 5 ng/µl frontalin and 40 ng/µl brocain respectively. The asterisks indicate the significance levels (*** = <0.001).

Blend A was the most attractive blend amongst all the six blend formulations. Comparison of *H. hampei* responses to solvent and individual components making up the blend showed that most insects highly preferred the blend than the solvent control and the frontalin component, (+ 41.4%, χ^2^_1_ =  25.46, P =  < 0.001; +32%, χ^2^_1_ =  14.69, P  = < 0.001 respectively). However, brocain attracted significantly more borers than the blend (+28%, χ^2^_1_ =  11.31, P  = < 0.001) (figure 8).

### Petri dish assays with frontalin and brocain

**Figure 9:**
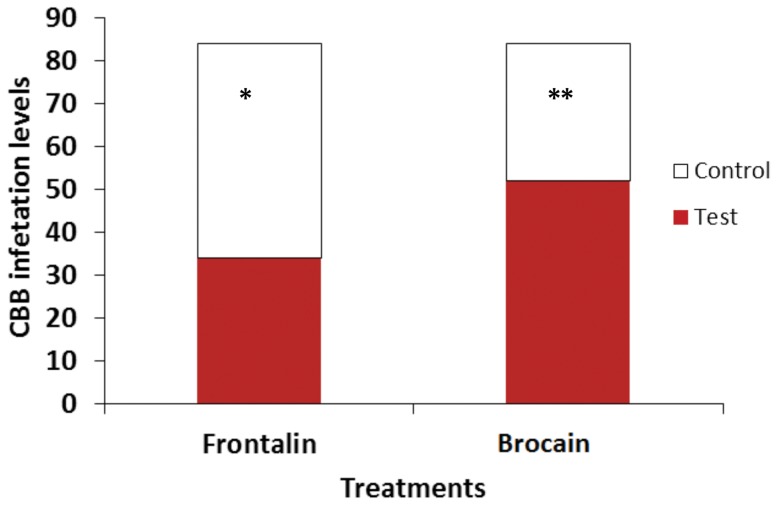
Comparison of *Hypothenemus hampei* (CBB) infestation levels of ripe coffee berries treated with either 80 ng/µl frontalin or 40 ng/µl brocain. The control is healthy berries treated with solvent (5% Dmso +95% water). The asterisks indicate the significance levels (* =  significant at 0.05 and ** =  significant at 0.01.

*H. hampei* successful colonization and infestation levels were significantly low in berries whose surface were treated with frontalin, compared to berries treated with solvent (control) (−26%, χ^2^_1_ =  4.62, *P*  = < 0.05). However, brocain appeared to enhance arrestment in *H. hampei* that led to higher infestation levels in brocain-treated berries than the control (solvent treated berries). Berries treated with 40 ng/µl brocain were significantly more attractive than the control (+30%, χ^2^_1_ =  7.41, P  = < 0.01) (figure 9).

**Figure 10:**
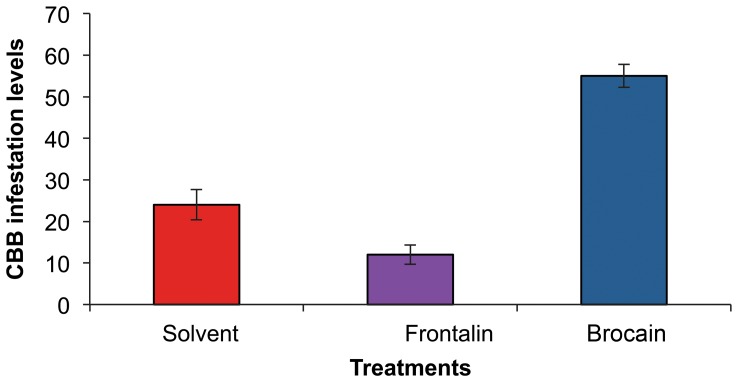
Comparison of *Hypothenemus hampei* (CBB) infestation levels of ripe coffee berries treated with solvent (5% Dmso+95% water), 80 ng/µl frontalin or 40 ng/µl brocain.

Likewise, in multiple-choice assays, there were differential colonization levels by females of berries treated with brocain, frontalin or solvent (figure 10). Significant differences in the levels of infestation among berries treated with either compound were recorded (χ^2^_2_ = 44.77, df = 2, *P* = <0.001). Frontalin disrupted infestation by 50% while brocain doubled infestation levels compared to the control.

### Field trials

**Figure 11:**
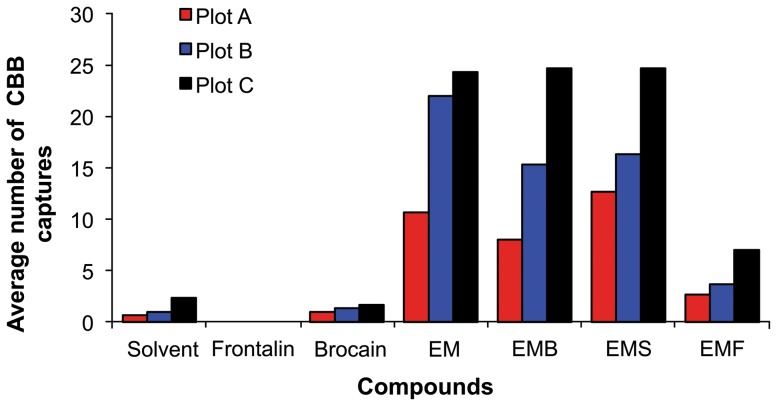
Number of coffee berry borer females (CBB) captured in traps baited with: brocain, frontalin, solvent, ethanol+ methanol mixture (EM) and blends of; ethanol: methanol + brocain (EMB); ethanol: methanol + frontalin (EMF) and ethanol: methanol + Solvent.

In general, low numbers of *H. hampei* females were trapped in the field assays (figure 11). Five hundred and forty (540) female borers were captured in all traps during the trial period. Trap captures varied significantly among the seven treatments (*F*_ 6,9_ = 22.26, *P* = <0.0001) (table 2). Traps baited with brocain trapped the lowest number of females, which did not vary significantly from the control trap. Traps baited with frontalin trapped no insects. The highest number of females was recorded in traps baited with *H. hampei* commercial attractant, the 1∶1 ethanol: methanol (EM) mixture. However, when the EM mixture was combined with frontalin, a significantly fewer *H. hampei* were caught in the traps compared to traps baited with EM lure alone and EM lure mixed with either solvent or brocain.

**Table 2:** Average (± SE) *Hypothenemus hampei* captures of traps baited with different compounds.

Compound	Average *H. hampei* captures
Ethanol: Methanol	19.00±4.29 a
Ethanol: Methanol + brocain (EMB)	17.89±4.13 a
Ethanol: Methanol + solvent (EMC)	16.00±4.37 a
Ethanol: Methanol + frontalin (EMF)	4.44±1.32 b
Brocain	1.33±0.24 b
Control	1.33±0.33 b
Frontalin	0.00±0.00 c

*Means followed by the same letter are not significantly different (*p = *0.05, SNK test).*

Furthermore, *H. hampei* captures were significantly different among plots (*F*_2,21_ = 5.27, *P* = <0.01) (table 3). Plot C with full sunlight exposure had the highest *H. hampei* female captures whereas plot A, which was adjacent to a shaded plot had the lowest number of captures.

**Table 3:** Average (± SE) *Hypothenemus hampei* captures in the three coffee plots.

Plots	Average *H. hampei* captures
A	5.10±1.53 d
B	8.52±2.43 de
C	12.10±3.12 e

*Means followed by the same letter are not significantly different (*p = *0.05, SNK test).*

## Discussion

Results from our behavioural assays show that (*5S,7S*)-conophthorin, frontalin and brocain appear to play an important role in the host discrimination and colonization processes of *H. hampei*. These findings are supported by our chemical analysis of volatiles which showed quantitative changes in the levels of (*5S,7S*)-conophthorin and frontalin in the headspace volatiles of coffee berries at different stages of infestation by *H. hampei*. We were unable to quantify the emission rates of brocain in the headspace samples because of the very low quantities detected. A follow-up experiment combining several samples will enable better quantification of the amounts of brocain released. These two spiroacetals were identified as the main components emitted by coffee berries in the early stages of *H. hampei* infestation. The levels of these components varied depending upon the presence or absence of *H. hampei* immature stages in the berries and the number of colonizing females. When *H. hampei* females were exposed to volatile extracts that had been collected at different times after the beginning of the infestation process and in coffee samples that were infested with different numbers of colonizing females, we recorded significant attraction to samples that had the highest amounts of (*5S,7S*)-conophthorin. This attraction disappeared as the levels of (*5S,7S*)-conophthorin decreased in the samples where colonizing females were either crowded or in the advanced stages of infestation (15 and 30 days post-infestation). A previous study had shown that conophthorin attracted the coffee berry borer [12]. Thus, we hypothesize that coffee berries producing high levels of conophthorin would likely exert a ‘pull’ effect on the coffee berry borer. Interestingly, conophthorin is widely known to repel conifer inhabiting bark beetles either as a pheromone or a non-host volatile component of some angiosperm trees [23]–[25], but is an attractant for two *Pityphthorus spp* [26]. Similarly, our results indicate that like *Pityphthorus spp, H. hampei* uses conophthorin as an attractant.

We also found that frontalin levels significantly increased in the volatiles of early infestation stages of berries by two *H. hampei* females, which significantly decreased in the advanced infestation stages, as well as berries infested by 4 and 6 colonizing females. Moreover, olfactory assays with frontalin elicited avoidance in *H. hampei*, and frontalin significantly reduced the pull of an attractive dose of brocain while tested in blends. This suggests that frontalin potentially serves as a repellent and elicits avoidance in conspecifics. In Petri dish assays only half of *H. hampei,* infested frontalin-treated berries compared to the solvent-treated berries. Interestingly, frontalin is an aggregation pheromone in economically important bark beetles [27]. These studies and our findings suggest that different insects use the same semiochemical for different behavioural activities.

In our study, brocain was attractive at a low dose but repellent at high doses, suggesting a dose-dependent dual function of brocain. Such dual purpose functioning of semiochemicals has been reported in various bark beetle species [28]–[30].

The fact that our results suggest an attractant function for brocain and conophthorin, while frontalin is a repellent in *H. hampei*, following the opposite mechanism found in other conifer-attacking Scolytidae, further supports the hypothesis by Jaramillo et al. [12], that angiosperm- attacking scolytids use host and non-host volatiles to navigate mixed forests.

The results from our field assays with different lures confirmed our laboratory findings that frontalin caused a repellent or avoidance behaviour in females of *H. hampei*. When we tested a commercial attractant comprising a 1∶1 mixture of ethanol and methanol [7] combined with frontalin, trap captures decreased by 77% suggesting that frontalin acts both as a repellent and an inhibitor of *H. hampei* attractants (brocain and methanol: ethanol). This implies that the presence of frontalin in the headspace volatiles is crucial in the host discrimination and colonization processes of *H. hampei* compared to the common fermentation product methanol and ethanol. This observation is supported by other reports of the inhibitory effect of anti-aggregation pheromones and repellent host compounds to attractants in various *Ips* and *Dendroctonus* species e.g [16], [31]–[34].

GC/MS analyses indicated quantitative differences in the levels of frontalin in berries in different infestation stages. Our behavioural assays confirmed differential responses of *H. hampei* to different doses of frontalin. Thus, we propose that frontalin may play a dual function in the chemical ecology of the coffee berry borer; as a defensive compound emitted by the coffee berry to protect itself from herbivory and also as a host marker (repellent) used by *H. hampei* to space its population in order to avoid competition from conspecific colonizing females. More research is needed to confirm this proposition. In some species in the Coleoptera, Lepidoptera, Diptera and Hymenoptera [35]–[40], host marking with a chemical is an important survival factor, preventing other females from exploiting the marked host to lay eggs or compel competitors to lay fewer eggs [41]. In this study, *H. hampei* female egg laying response matched with previously observed egg laying response in the presence of competitors, whereby berries with high numbers of colonizing females were the least reproductive.

It is known that a coffee berry is usually attacked by a single *H. hampei*. Since a single berry may accommodate up to approx. 200 eggs of the pest [42], this may reflect an adaptive behaviour of the colonizing females to repel incoming colonizing beetles. It appears that the limited carrying capacity of the berry, as the niche would only suffice for its brood to complete the life cycle. Attack density regulation is common among bark beetles due to its effect on reproduction and population dynamics [43]–[44]. Thus, the colonizing females may detect the density of conspecifics amongst potential hosts before landing depending on the type and concentrations of semiochemicals released by the beetles/plant already attacking a host [10], [45]. As previously indicated, *H. hampei* attraction was only observed to berry volatiles infested by 2 *H. hampei* per berry in early the infestation (only eggs stages present) while avoidance or repellence was observed in berries infested by 4 and 6 initial colonizing females per berry, and late infestation stages (all life stages present). This avoidance and lack of host recognition behaviour of the females coincided with diminished amounts of conophthorin, which is the pest attractant [12]. Kraker [6] also reported that borer-infested berries caused avoidance behaviour in *H. hampei*. It therefore appears that *H. hampei* has a mechanism that limits pioneer females from sharing a host, through release of repellent compounds and/reducing production of attractants by the host, such that approaching beetles are repelled or fail to recognize the host. Our findings suggest that frontalin could serve both as a repellent and a spacing factor in *H. hampei*, as has been reported for various bark beetles [8]–[10], [45].

A previous study [14] reported that frontalin could play a role as a spacer pheromone of some bark beetles. Its production has been reported to contribute to terminating aggregation behaviour in the mountain pine beetle, *Dendroctonus ponderosae* by signalling approaching conspecifics of the unavailability of enough food in the already attacked host trees [13], [15]. More recently, Liu et al. [17] reported that frontalin acted as both as an aggregation pheromone and a sex pheromone for *Dendroctonus valens*, although high concentrations were found to reduce female attraction.

In our field study, the lowest *H. hampei* trap catches were recorded in the plot adjacent to the shaded plot, while the highest captures were recorded in the traps located in the sunny areas. The variation in the trap captures in the different plots is an interesting finding, which would require extensive replication. Previous studies reported that intercropping coffee with shade trees reduced *H. hampei* infestation levels [12], [46], [47]. We suspect that semiochemical diversity, ratios and concentrations in a mixed coffee cropping system may all contribute to minimizing pest populations.

In summary, this study has investigated the contribution of frontalin and brocain to the host colonization process in *H. hampei*. Taken together, with results from our previous studies, we now know the role of conophthorin, brocain and frontalin in the chemical ecology of *H. hampei*. From the perspective of pest management, the three spiroacetals could be potential candidates for coffee berry borer management in a ‘push pull’ strategy whereby; frontalin acts as the ‘push’ (repel) from the host with conophthorin or brocain acting as the ‘pull’ (attractant).

## Supporting Information

Figure S1 **Release rate (ng/µl/hr) of (** ***5S,7S*** **)-conophthorin (purple) and frontalin (orange) of berries (approx. 150 days of development) at different stages of infestation by** ***Hypothenemus hampei*** **.**
(TIF)

Table S1 **Release rates of compounds tested during field trials.**
(DOCX)

Table S2 **Statistics on effect of evaluation time on individual** ***Hypothenemus hampei*** **life stages.**
(DOCX)
