# Supplementary figures and images for: Spiroacetals in the Colonization Behaviour of the Coffee Berry Borer: A ‘Push-Pull’ System
Source: PLoS One. 2014 Nov 7;9(11):e111316. doi: 10.1371/journal.pone.0111316 (PMC4224388; doi:10.1371/journal.pone.0111316)

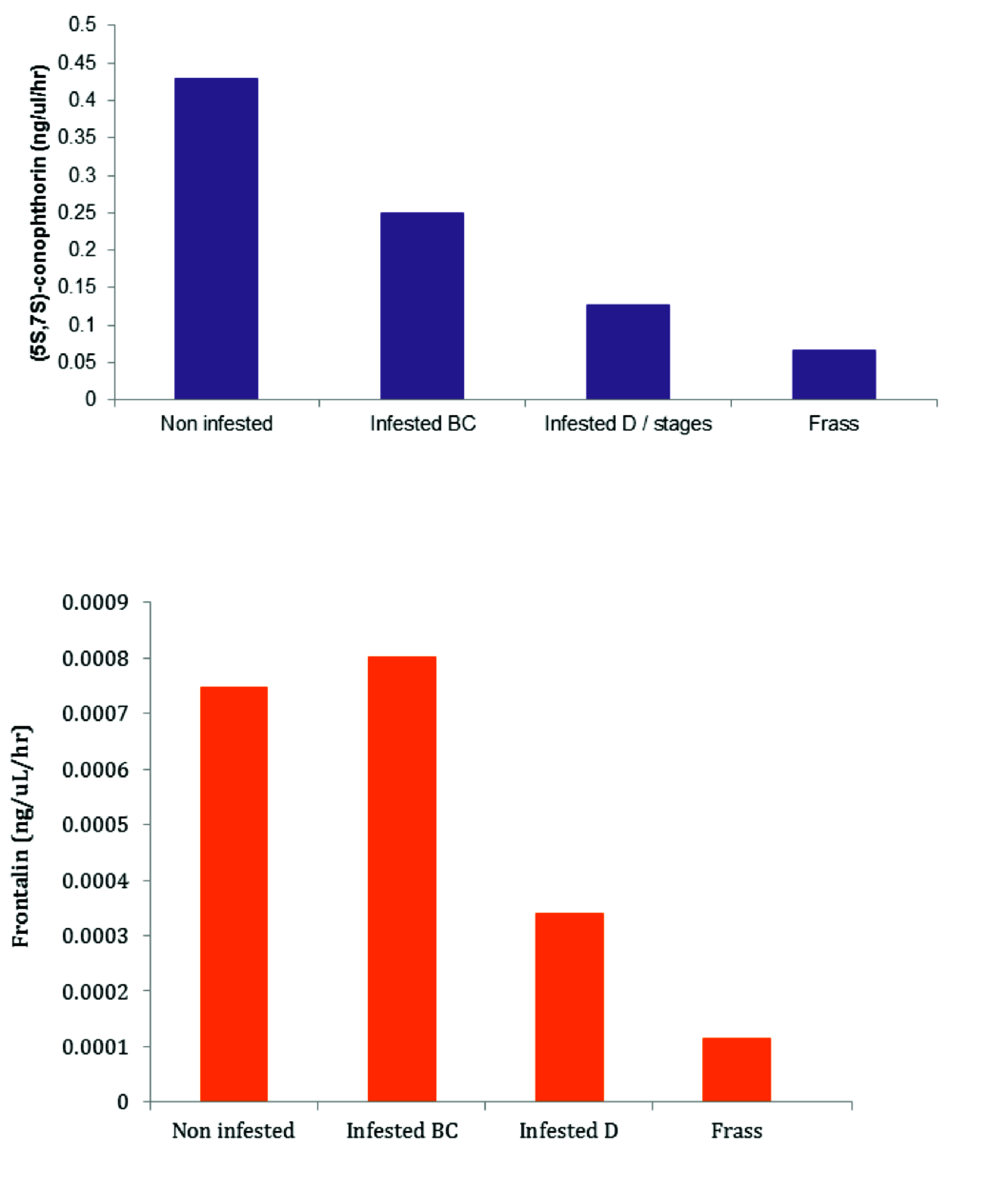

Supplement: Figure S1 — Release rate (ng/µl/hr) of ( 5S,7S )-conophthorin (purple) and frontalin (orange) of berries (approx. 150 days of development) at different stages of infestation by Hypothenemus hampei . (TIF) [file pone.0111316.s001.tif]
