# Supplementary material for: Spiroacetals in the Colonization Behaviour of the Coffee Berry Borer: A ‘Push-Pull’ System
Source: PLoS One. 2014 Nov 7;9(11):e111316. doi: 10.1371/journal.pone.0111316 (PMC4224388; doi:10.1371/journal.pone.0111316)
Supplement: Table S1 — Release rates of compounds tested during field trials. (DOCX) [file pone.0111316.s002.docx]

Table S1. Release rates of compounds tested during field trials

| Compounds tested in traps | Average release rates (µl/day) | |
| --- | --- | --- |
| Brocain | 66.67 ±4.7 |  |
| Frontalin | 80.95±9.5 |  |
| Methanol+ Ethanol | 166.67 ±23.8 |  |
| Solvent (95% H20+ 5% DMSO) | 100 ± 21.8 |  |
| Methanol+ Ethanol+ Brocain | 189.52±12.60 |  |
| Methanol+ Ethanol+ Solvent | 341.90±26.5 |  |
| Methanol+ Ethanol +Frontalin | 251.43±21.82 |  |
