# Supplementary material for: Spiroacetals in the Colonization Behaviour of the Coffee Berry Borer: A ‘Push-Pull’ System
Source: PLoS One. 2014 Nov 7;9(11):e111316. doi: 10.1371/journal.pone.0111316 (PMC4224388; doi:10.1371/journal.pone.0111316)
Supplement: Table S2 — Statistics on effect of evaluation time on individual Hypothenemus hampei life stages. (DOCX) [file pone.0111316.s003.docx]

Table S2. Statistics on effect of evaluation time on individual *Hypothenemus hampei* life stages

| Stage | Evaluation time (days) | Number of *H. hampei* colonizing females per berry | | | Test statistic |
| --- | --- | --- | --- | --- | --- |
|  |  | 2 | 4 | 6 |  |
| Eggs | 2 | 0.094 | 0.036 | 0.035 | F_2,141_=1.20, P=0.3044 |
|  | 5 | 0.479 | 0.453 | 0.281 | F_2,141_=0.65, P=0.5256 |
|  | 15 | 3.969 | 2.469 | 2.007 | F_2,141_=5.19, P=0.0067 |
|  | 30 | 1.198 | 0.500 | 0.531 | F_2,141_=4.69, P=0.0107 |
|  |  |  |  |  |  |
| Larvae | 2 | 0.000 | 0.000 | 0.000 | _ |
|  | 5 | 0.031 | 0.000 | 0.000 | F_2,141_=1.00, P=0.3705 |
|  | 15 | 2.625 | 1.510 | 1.417 | F_2,141_=4.35, P=0.0146 |
|  | 30 | 4.823 | 3.172 | 3.226 | F_2,141_=4.03, P=0.0199 |
|  |  |  |  |  |  |
| Pupae | 2 | 0.000 | 0.000 | 0.000 | _ |
|  | 5 | 0.000 | 0.000 | 0.000 | _ |
|  | 15 | 0.000 | 0.000 | 0.000 | _ |
|  | 30 | 1.708 | 1.104 | 0.899 | F_2,141_=6.24, P=0.0025 |
|  |  |  |  |  |  |
| Adult | 2 | 0.000 | 0.000 | 0.000 | _ |
|  | 5 | 0.000 | 0.000 | 0.000 | _ |
|  | 15 | 0.000 | 0.000 | 0.000 | _ |
|  | 30 | 1.844 | 1.354 | 1.313 | F_2,141_=2.79, P=0.0647 |
